# Necrotizing Fasciitis of the Breast Requiring a Life-Saving Mastectomy: A Case Report and Literature Review

**DOI:** 10.7759/cureus.19886

**Published:** 2021-11-25

**Authors:** Shariful Islam, Imran Aziz, Jitendra Shah, Vijay Naraynsingh, Patrick Harnarayan

**Affiliations:** 1 General Surgery/Oncoplastic Breast Surgery, San Fernando General Hospital, San Fernando, TTO; 2 Department of Clinical Surgical Sciences, University of the West Indies, St. Augustine, TTO; 3 Surgery, San Fernando General Hospital, San Fernando, TTO; 4 Department of General Surgery/Breast Surgery, San Fernando General Hospital, San Fernando, TTO; 5 Surgery, Medical Associates Hospital, St. Joseph, TTO; 6 General Surgery, San Fernando General Hospital, San Fernando, TTO

**Keywords:** necrotising fasciitis of the breast, insect bites, diabetes, breast, gas gangrene

## Abstract

Necrotizing soft tissue infection of the breast is an extremely rare event in routine surgical practice. It is the most aggressive form of soft tissue infection and a real surgical emergency. It is associated with a high risk of mortality if not diagnosed promptly. A Literature search has revealed only a few such cases. The exact etiology is variable and very often multifactorial. Early recognition and prompt surgical treatment along with broad-spectrum antibiotic therapy are of paramount importance to prevent mortality. In this report, we present the first case of necrotizing fasciitis of the breast following an insect bite in the literature, in a 57-year-old diabetic patient with a delayed presentation that required a life-saving mastectomy.

## Introduction

Necrotizing soft tissue infection is a rare but potentially fatal disease [1]. It is one of the most severe and aggressive forms of soft tissue infections. The initial presentation varies from minor/mild soft tissue infections to severe forms of septic shock and multi-organ dysfunction syndrome [2]. It frequently affects the perineum, abdominal wall, and extremities; however, it can occur anywhere on the body, including, in very rare cases, the breasts [1]. Necrotizing soft tissue infection of the breast can be idiopathic or occurs secondary to a causative agent. It is usually seen in diabetic or immunocompromised patients, but it has also been reported in healthy patients. It is associated with a mortality rate as high as 73%; however, it can be reduced to 10% with the appropriate treatment [3]. Early recognition, as well as prompt surgical treatment, is of paramount importance. Delayed presentation, as well as misdiagnosis, often leads to a total mastectomy, which has profound physical and psychological consequences or life-threatening outcomes [4,5]. To our knowledge, this is the first case of insect bite-associated necrotizing soft tissue infection of the breast to be reported in the literature.

## Case presentation

**Figure 1:**
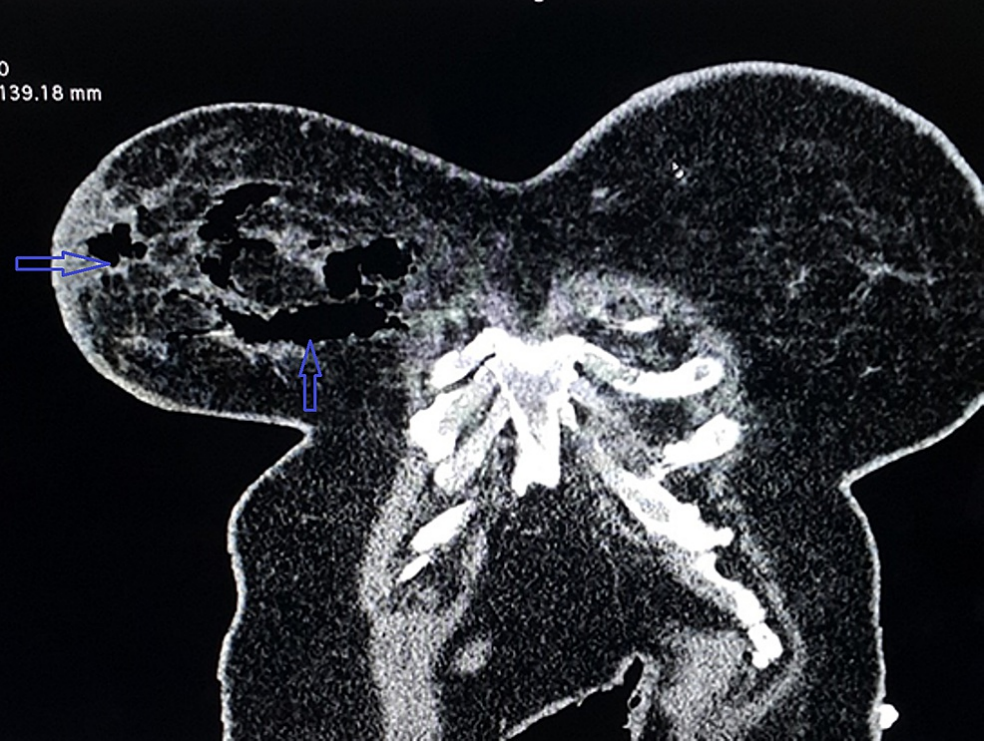
CT pulmonary angiogram showing large pockets of gas within the right breast parenchyma (blue arrows) with edema of the overlying skin CT: computed tomography

A 57-year-old diabetic, hypertensive, and obese female was admitted to the medical ward at the General Hospital for sudden right-sided chest pain for one day. A CT pulmonary angiogram was performed, which reported no pulmonary embolism; however, there was a large pocket of air within the right breast suggestive of gas gangrene of the right breast with significant edema within the overlying skin (Figure 1). The patient's condition worsened over the next few hours with increased right breast pain, swelling, shortness of breath, and ruptured bullae at the 9 o'clock position of the breast. She was immediately referred to the general surgeons; on further scrutiny, she reported having been bitten by an insect in the inferior aspect of the breast two weeks prior but stated that she had not taken it seriously She also reported a family history of breast cancer, with two close relatives having the disease.

On clinical examination, she was found to be tachypnoeic, and tachycardic with a rate of 130 beats per minute; however, she was afebrile but her overall general appearance was one of a toxic-looking patient. The right breast was found to be swollen, tender, and with discolored necrotic skin at the 9 o'clock position. Her laboratory tests showed a white cell count of 17.56 x 10^9^/L with mild left shift and a normal hemoglobin level of 12 gm/dl; her renal serum electrolytes were within normal range but her serum creatinine was elevated at 3.5. An arterial blood gas identified a metabolic acidosis with a pH of 7.30 and lactate of 7.4 mmol/L. Creatinine was elevated at 394 umol/L, and broad-spectrum antibiotics were started. After prompt resuscitation, the patient consented to undergo emergency debridement of the right breast.

**Figure 2:**
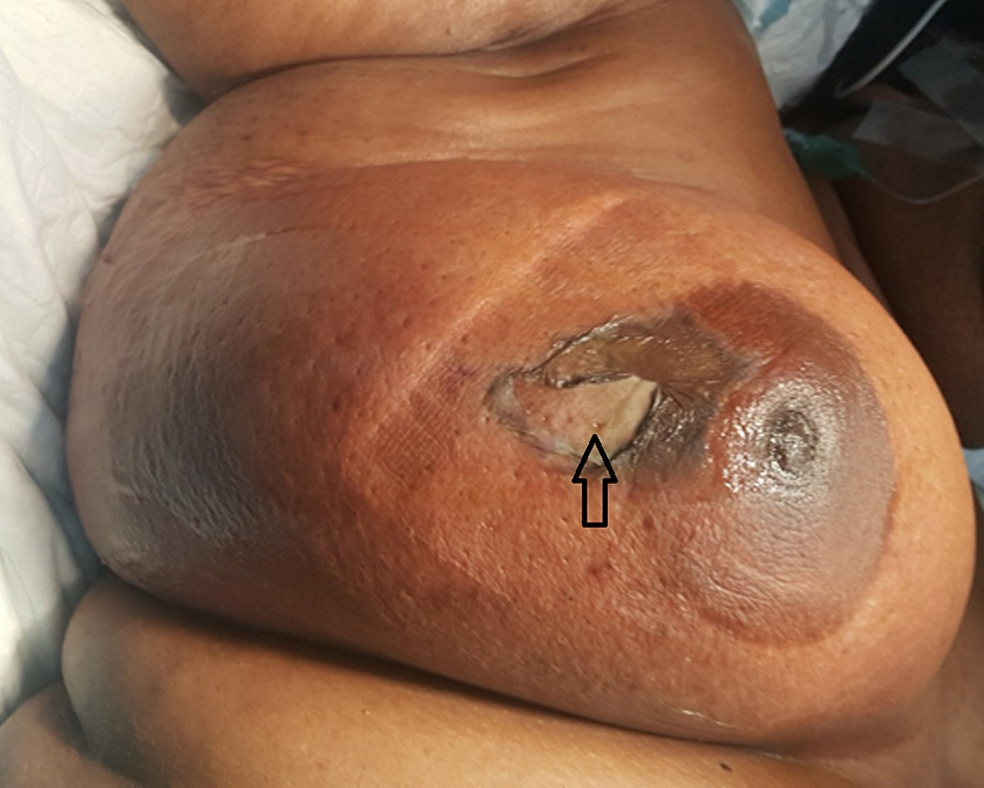
Picture showing grossly edematous right breast with gangrenous patches of skin at 9 o'clock position close to nipple-areolar complex (black arrow indicates the site of insect bite)

**Figure 3:**
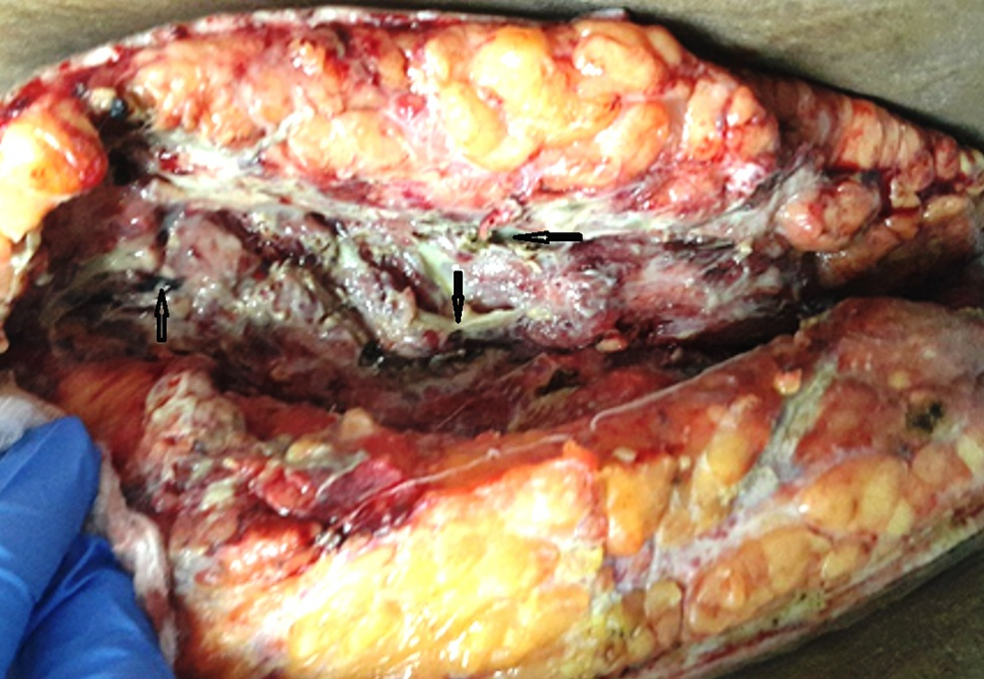
Intraoperative photograph showing gangrenous breast tissue and fasciae (black arrows)

The surgical findings included the presence of air within the breast with almost 100% of the breast being necrotic with dead overlying skin; a mastectomy was performed. The finger test was used when the pectoral fascia was encountered at the depth of the wound; this showed no further spread across the chest wall. With most of the breasts removed to achieve healthy margins, the wound was packed with gauze (Figures 2, 3).

**Figure 4:**
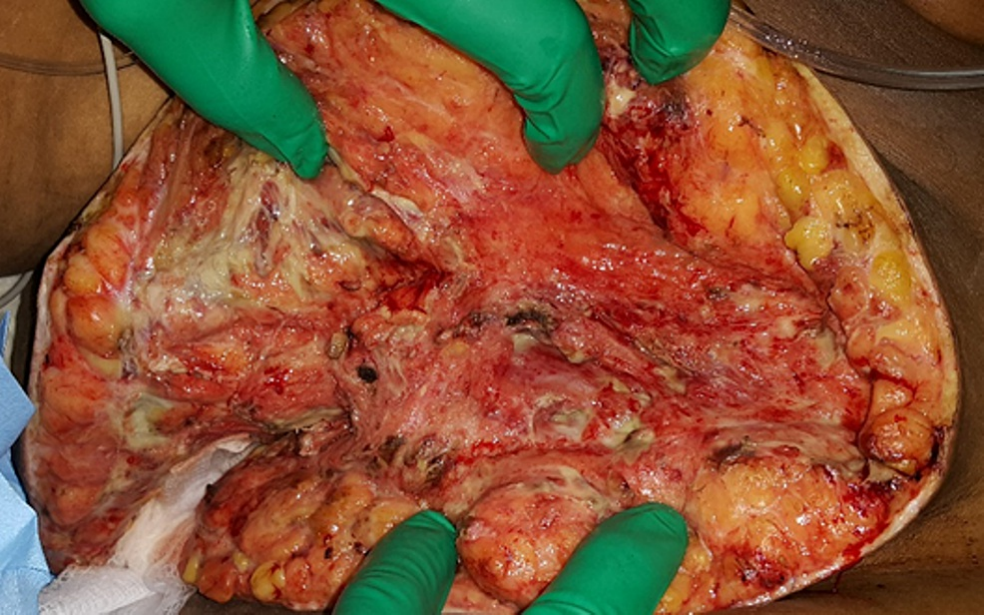
Postoperative photograph showing good granulation tissue with minimal slough at the base

Gram staining of a representative sample of breast tissue was performed and found to contain only Gram-negative bacteria. Physiologically, however, our patient became acidotic and hypotensive and required noradrenaline support. She was transferred to the ICU. Her stay at the ICU was uneventful, and she was slowly weaned off pressure support and discharged to the high dependency unit (HDU) on day seven. Her microbiology report demonstrated *Klebsiella pneumoniae* species sensitive to carbapenem. The wound was examined daily for the first few days and found to be healthy-looking with minimal slough at the base (Figure 4). A vacuum dressing was applied later on to assist in the closure of the wound, and delayed closure of the wound was done.

**Figure 5:**
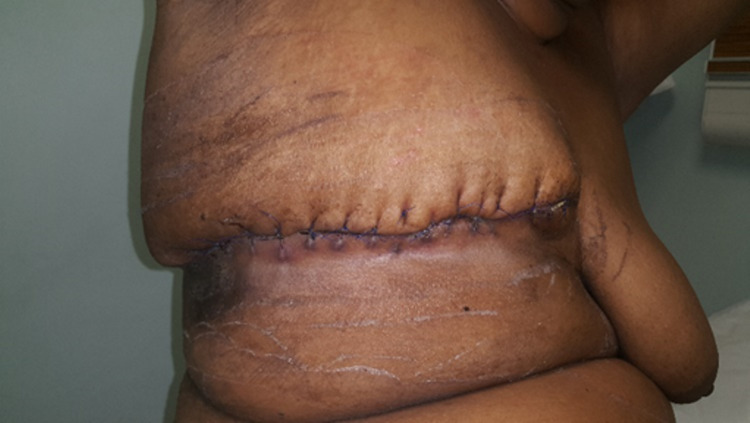
Photograph showing healing of the wound after emergency mastectomy

The patient was discharged on the 21st postoperative day. She was followed up in the surgical outpatient clinic and her wound healed well. Final histology did not reveal any malignancy; however, it showed extensive necrosis of the skin, fascia, and breast tissue with neutrophilia in the necrotic area. At the one-year follow-up at the surgical clinic, she was found to be doing well with no further complaints (Figure 5).

## Discussion

Necrotizing soft tissue infections are a severe and aggressive form of infection. The exact nature of this clinical entity remains undiscovered and uninvestigated. It is often considered a Fournier type of gangrene complicated by obliterative arteritis. It commonly affects the perineum, abdomen, and extremities, and, in rare cases, the breast. In the breast, it is usually a unilateral rather than a bilateral infection.

Despite advancements in technology and the use of broad-spectrum antibiotics, several studies still suggest only a minimal reduction in the mortality rate, from 35 to 23.5% [2,3]. The high mortality and morbidity rate can be attributed to delays in the diagnosis and institution of appropriate therapy, which is not only a reflection of its rarity but also a lack of familiarity on the part of the physicians. The condition is commonly reported in patients who are immunocompromised, those with diabetes mellitus [6], HIV infection [7], intravenous drug use, and alcoholism. It is also reported in non-lactating healthy patients [2,5,8-12].

Necrotizing fasciitis of the breast can be idiopathic [1,13] or secondary to other causes, i.e, following a needle core biopsy [14,15], elective total or partial mastectomy [7,16-19], human bites [15], penetrating injury [20], anticoagulation therapy [21-23], topical belladonna application [24], in patients with puerperal sepsis [25], and breast tumors with mammary infarcts in pregnant patients [26,27]. Our literature search revealed that trauma, small trivial wounds, or even minor scratches can cause necrotizing fasciitis [28]. Our patient was diabetic and described an insect bite on her right breast. This, in conjunction with her delayed presentation, may have led to the necrotizing infection of her right breast.

Based on the type of organism isolated, necrotizing infections can be classified into two types. Type 1 is polymicrobial and consists of Gram-positive, Gram-negative bacteria, anaerobes with a fulminant course of the disease [9,14]. The type 2 variant is caused by group A *Streptococcus* and is frequently found in high-risk patients [29], i.e, diabetic, obese, immunocompromised [14], and patients with peripheral vascular disease. The causative organism in our patient was *Klebsiella pneumoniae* and it was sensitive to carbapenem.

The diagnosis is usually made according to a combination of clinical and surgical findings. However, various imaging modalities, i.e., ultrasonogram (USG), CT, and MRI scans are also used to aid the diagnosis [14]. Ultrasound findings usually reveal edematous thickening of the fascia associated with fluid collections [14]. CT scan can demonstrate the presence of subcutaneous gas and fat stranding. Kaczynski et al., in 2012, first reported gas in the subcutaneous tissue of the breast in a 75-year-old patient [30]. The CT scan of our patient revealed large pockets of gas within the breast parenchyma. Although MRI has a low specificity of 46-86%, it can reveal the extent of deep tissue involvement in more detail than any other imaging modality [14,31,32]. Even though imaging is a useful adjunct in establishing the diagnosis, it should not cause delays in delivering definitive care.

Successful surgical outcome typically involves a multidisciplinary approach. An ideal approach should involve early diagnosis, aggressive resuscitation with intravenous fluids, antibiotics, and intensive care support along with serial radical debridement of all non-viable tissues [33].

Most reports in the literature suggest staged debridements rather than immediate mastectomy as a treatment strategy. Although breast conservation is possible in most cases [11,30], early radical mastectomy can be life-saving in severe cases of necrotizing fasciitis [2,8,9], as was the case with our patient. If the defect is large and not amenable to primary closure, a vacuum-assisted closure (VAC) dressing can assist in the closure of the wound [20] but may often require a split-thickness skin graft to cover the defect [34] or other advanced reconstructive procedures [4,9].

Two recent systematic reviews of primary necrotizing fasciitis of the breast have noted that 52.5% of the cases were idiopathic and 37.5% were secondary following surgery. Mixed organisms were commonly identified in 42.5% of cases compared to a single organism (*Streptococcus pyogenes*) in 37.5% of cases. Total or radical mastectomies were performed in 52% of cases for source control. Excisional debridement and partial mastectomy accounted for 32% and 12% of the cases respectively. Split-thickness skin grafts (44.4%) and delayed primary closures (33.3%) were the most common methods of reconstruction. Septic shock at presentation was an independent risk factor for mortality (12.5%) in these patients (p<0.001) [35,36].

## Conclusions

We reported the first case of an insect bite in a diabetic patient causing life-threatening gas gangrene of the breast requiring emergency life-saving mastectomy. Breast gangrene is a very rare entity and its exact etiology is variable and very often multifactorial. Iatrogenic trauma is a known risk factor in immunocompromised patients; however, it is also reported in healthy lactating mothers with teeth bites or due to unsterilized needle application to the breast. Insect bites can be an inciting factor, although it is not yet documented in the literature. Delayed presentation is very detrimental and often results in unfavorable cosmetic outcomes. Diagnosis is confirmed on the basis of clinical and laboratory findings, imaging, and culture findings. Prompt and serial surgical debridement along with broad-spectrum antibiotic treatment continues to be the gold standard in the management of these patients and is crucial for a better prognosis. Most of the cases with primary necrotizing fasciitis of the breast are managed with a mastectomy to gain source control. Closure can be easily achieved by delayed primary closure, local tissue rearrangement, VAC, full-thickness skin grafts, and pedicle flap reconstruction. However, reconstruction should be individualized, patient-dependent, and should follow the reconstructive ladder.
